# Retroversion of the Gravid Uterus—Retention of Urine—Recovery

**Published:** 1875-07-01

**Authors:** Arthur W. Edis


### RETROVERSION OF THE GRAVID UTERUS—RETENTION OF URINE—RECOVERY.

By Dr. Arthur W. Edis.

*From Obstetrical Journal of Great Britain and Ireland, June, 1875.*

C. G., aged twenty-five; married five years; mother of two children, youngest two years. Last catamenia three months since. Presented herself in the out-patient department on December 19th, complaining of severe pain in the lower abdomen, with swelling, and inability to hold or pass her water, there being also much difficulty in getting the bowels to act, with much bearing-down pain. The patient was unable to lie on either side, and had been unable to do any work for the last three weeks.

About the beginning of December, on attempting to pass water one evening, she found she was unable; but during the night and the following day, "it came so quick she could not stop it." And since then she had been in a constant state of discomfort from the continuous flow of urine. On examination *per vaginam* the pelvis was found to be blocked up by an enlarged retroflexed gravid uterus, at about the third month of utero-gestation. The fundus was low down, pressing on the perineum, and causing a distinct rectocele. The os was hardly to be felt, high up beyond the pubis, above which, externally, a tense, firm, resisting swelling, with indistinct evidence of fluctuation, occupying the centre of the abdomen, and extending up beyond the umbilicus, was detected.

A No. 8 elastic catheter was passed with some difficulty in a vertical direction, and 94 ounces of turbid, ammoniacal urine were drawn off. The patient was then supported in the knee-shoulder position, two fingers of the left hand were passed per rectum, and the fundus guided round the right side of the promontory of the sacrum, the os descending to the normal position, lowest in the pelvis. A Hodge was inserted, and the patient instructed to relieve the bladder frequently. Ergot, strychnia, and bark were prescribed.

On the 21st she stated that the difficulty in holding her water had returned at 11 P.M. on the 19th, and the urine had run away from her ever since, the Hodge having been forced out the following day. On examination the condition of affairs was the same as when first seen, 86 ounces of turbid urine were drawn off, and the patient lying on the left side, the uterus was replaced as before, and a larger Hodge inserted.

On the 23d she stated that she had had perfect control over the bladder since the last examination. The uterus was now found to be in its normal position, the os being lowest. Twelve ounces of clear, healthy urine were drawn off to see if there were any further retention; the Hodge was *in situ*.

On the 30th she was seen, and was perfectly comfortable, there being no return of the difficulty in micturition. Hodge *in situ*. She has been seen twice since this, and remains convalescent. Pregnancy advancing normally.

---

TREATMENT OF ABSCESS OF BREAST BY COMPRESSED SPONGE.—A patient had been suffering from mammary abscess for three weeks, but without any special benefit from treatment in checking the discharge of pus. It was decided to try the effect of compressed sponge, and for this purpose a sponge about ten inches in diameter was subjected to pressure and then applied by means of a bandage over the breast. After it had been in use forty-eight hours the abscess was completely cured. No pain was experienced by the patient, and in this case the opening in the breast was three inches above the dependent part of the abscess. In applying a sponge to the breast in this class of cases, it is found of advantage to compress it when dry. After it is applied to the breast and firmly secured in position, a little water is poured upon it to cause expansion and the necessary pressure.—*Ibid.*

---

CESAREAN SECTION; MOTHER AND CHILD SAVED.—Dr. Valentinotti reports a case of Cesarean section which was successful for the mother and child. The great interest of the case consisted in the use of an India-rubber cord covered with silk, such as is found in commerce, for the sutures. Four points of sutures, passing through the entire thickness of the uterine walls, were used. These threads were cut near the knots, and the remainder abandoned in the abdomen. The abdominal parietes were brought together with ordinary sutures. On the thirty-first day after the operation the patient walked about her room. A second interesting feature of the case was the use of chloral for diminishing the pain of the operation. Ten grammes were given in two doses, with an interval of about half an hour. The patient scarcely knew that the operation was taking place, and at its completion slept profoundly.—*Gazz. Obstet.* and *Giorn. Veneto di Scienz. Med.*, June, 1874.—*Ibid.*

---

DIAGNOSIS OF OVARIAN DISEASE.—Mr. Spencer Wells called attention, at a recent meeting of the London Pathological Society, to the fact that while extra-ovarian cysts are often radically cured by a single tapping, the cyst contracting and never refilling, true ovarian single cysts are almost certain to fill again. The contents of parovarian cysts consisted of little more than pure water.

